# Extending the Indications for Full Revascularization with Robotic-Assisted Coronary Artery Bypass

**DOI:** 10.3390/jcdd13090411

**Published:** 2026-08-24

**Authors:** Gökhan Arslanhan, Murat Bastopcu, Anıl Karaağaç, Halim Ulugöl, Muharrem Koçyiğit, Sena Sert Şekerci, Aleks Değirmencioğlu, Şahin Şenay, Cem Alhan

**Affiliations:** 1 Department of Cardiovascular Surgery, School of Medicine, Acibadem Mehmet Ali Aydinlar University, 34638 Istanbul, Turkey; gokhan.arslanhan@acibadem.edu.tr (G.A.); 2 Department of Cardiovascular Surgery, Acibadem Altunizade Hospital, 34662 Istanbul, Turkey; 3 Department of Anesthesiology, Vocational School of Health Services, Acibadem University, 34638 Istanbul, Turkey; 4 Department of Cardiology, School of Medicine, Acibadem Mehmet Ali Aydinlar University, 34638 Istanbul, Turkey; 5 Department of Cardiology, School of Medicine, Haliç University, 34060 Istanbul, Turkey

**Keywords:** robotic cardiac surgery, robotic-assisted minimally invasive direct coronary artery bypass, multivessel coronary artery bypass, complete revascularization, left atrial appendage occlusion, coronary endarterectomy

## Abstract

Coronary artery bypass grafting via median sternotomy carries considerable morbidity, and minimally invasive robotic approaches have been increasingly performed for surgical revascularization of coronary arteries. We report our institutional experience in robotic-assisted minimally invasive coronary revascularization in a broad patient population with complex multivessel disease. We retrospectively reviewed robotic-assisted minimally invasive direct coronary artery bypass (RA-MIDCAB) procedures performed at our center between January 2022 and June 2026. Patient demographics, additional procedures and in-hospital outcomes were recorded. A total of 242 patients were included (mean age 63.4 ± 9.7 years; 31 (12.8%) female). Single-vessel bypass was performed in 25 (10.3%) patients; 212 (87.6%) patients underwent an operation on the arrested heart (mean cross-clamp time 66.9 ± 21.7 min) and 15 (6.2%) received an off-pump operation (mean CPB time in on-pump patients 155.7 ± 46.0 min). Full arterial revascularization was achieved in 42 (17.4%) patients; a bilateral internal mammary artery configuration was used in 9 (3.7%) patients. Coronary endarterectomy was performed in 16 (6.6%) patients and concomitant left atrial appendage (LAA) occlusion was performed in three (1.2%) patients. Epiaortic ultrasonography-guided clamp placement was performed in 27 (11.2%) patients with ascending-aortic plaque. In-hospital mortality occurred in two (0.8%) patients; no patient sustained a major neurological deficit, and the transfusion rate was 9.9%. Mean ventilation time was 4.0 (3.0–6.0) hours and mean intensive care unit stay was 22.6 ± 11.2 h. With careful planning and accumulated experience, the indications for robotic-assisted minimally invasive revascularization can be extended to include patients who require full-arterial revascularization, have ascending aortic plaques, complex coronary disease requiring endarterectomy, or atrial fibrillation where concomitant left atrial appendage occlusion is indicated.

## 1. Introduction

Coronary artery disease remains the leading cause of death worldwide, and coronary artery bypass grafting (CABG) is the most commonly performed cardiac operation [1]. Candidates for CABG are typically identified by coronary angiography and evaluated by a multidisciplinary heart team before proceeding to surgery [2]. The conventional approach via median sternotomy is associated with considerable morbidity, including prolonged recovery and sternal wound infection [3]. Minimally invasive direct coronary artery bypass (MIDCAB) through a small left anterior thoracotomy was introduced as a sternal-sparing alternative for left internal mammary artery (LIMA) to left anterior descending artery (LAD) grafting [4]. In the randomized MIST trial, multivessel minimally invasive CABG through a small thoracotomy improved physical recovery at one month, with safety at one year comparable to CABG via sternotomy [5]. Robotic assistance for mammary artery harvest further refines this approach, offering high-definition three-dimensional visualization and enhanced dexterity that enable harvesting of both mammary arteries with reduced chest wall trauma [6,7,8]. Robotic-assisted minimally invasive coronary revascularization has been associated with reduced postoperative blood loss, shorter ventilation time and hospital stay, lower transfusion and wound complication rates, and improved cosmesis [9,10].

A principal limitation of these minimally invasive strategies has been their confinement to single- or double-vessel disease involving anterior targets. Complete coronary revascularization requires exposure of multiple coronary territories through a limited thoracic incision [11]. Consequently, robotic-assisted MIDCAB (RA-MIDCAB) has traditionally been reserved for isolated proximal or mid-LAD disease unsuitable for percutaneous coronary intervention, or for the surgical component of hybrid coronary revascularization in which non-LAD targets are addressed by stenting [12]. Patient selection has been governed by concerns that multivessel disease requiring lateral, posterior, or inferior grafting would lead to increased operative times, translating into prolonged postoperative mechanical ventilation and higher transfusion requirements [13]. Aortic calcification and additional planned procedures, such as endarterectomy, have likewise been regarded as reasons to select against a minimally invasive approach [14,15]. Left atrial appendage (LAA) occlusion is also rarely combined with minimally invasive CABG due to technical complexity [16].

Advances in operative exposure and surgical instruments have extended these boundaries. The use of cardiopulmonary bypass (CPB) with cardioplegic arrest through a third intercostal space thoracotomy, combined with techniques that permit controlled rotation and exposure of the arrested heart, allows distal anastomoses to be performed at all coronary targets under direct vision in a stable, motionless field [17]. Coupled with robotic mammary harvest and detailed preoperative computed tomographic planning of cannulation strategy and graft configuration, this approach makes complete multivessel revascularization feasible without sternotomy. We report our experience with robotic-assisted complete multivessel coronary revascularization, with the aim of demonstrating the feasibility and safety of extending minimally invasive robotic surgery to this broader, more complex patient population.

## 2. Materials and Methods

The robotic-assisted MIDCAB cases performed in our institution between January 2022 and June 2026 were identified from the hospital database. The robotic-assisted multivessel MIDCAB technique began in our center in January 2022, and all procedures were performed using the da Vinci Xi system (Intuitive Surgical, Sunnyvale, CA, USA). Patient demographics, preoperative imaging, operative details (conduit types, numbers of distal and proximal anastomoses, and concomitant procedures including coronary endarterectomy and LAA occlusion), and in-hospital outcomes were recorded.

Our technique for RA-MIDCAB comprises robotic harvest of the LIMA and right internal mammary artery (RIMA) followed by complete revascularization through a left anterior minithoracotomy, performed with CPB and cardioplegic arrest.

### 2.1. Preoperative Planning with Computed Tomography

**Figure 1:**
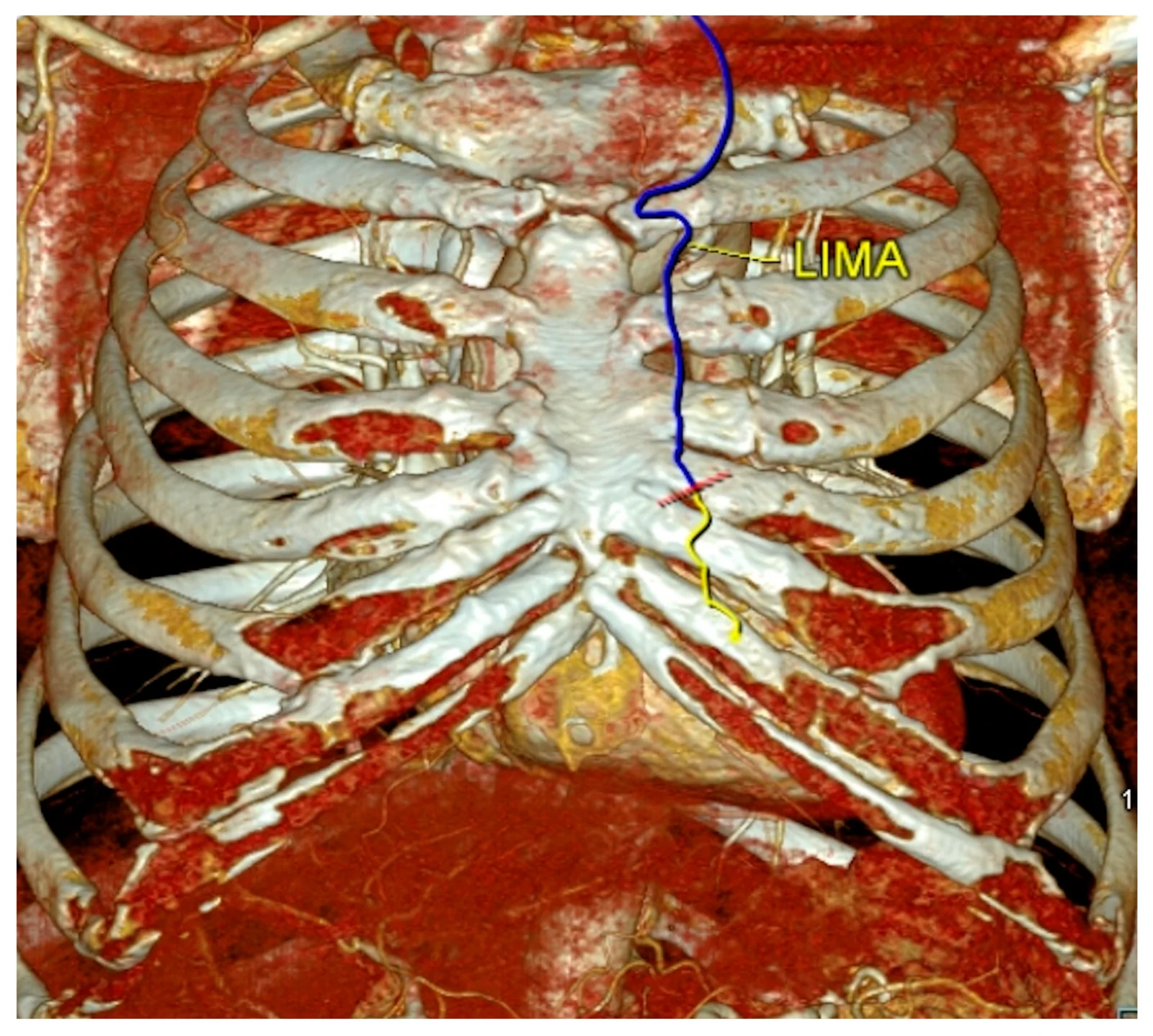
LIMA tortuosity and caliber are assessed using preoperative contrast-enhanced CT images.

All patients undergo a routine contrast-enhanced computed tomography (CT) scan before surgery. The descending aorta and the iliofemoral vessels are evaluated for calcification and thrombus, and the cannulation approach is selected accordingly. Any calcific or soft plaques in the ascending aorta are noted. The thoracotomy access site is likewise planned from the CT images. The intercostal space that is closest to the proximal ascending aorta is favored, as it facilitates manipulation of the aorta for proximal anastomoses and aortic root cannulation. The courses of the LIMA and RIMA are examined for tortuosity and caliber [18] (Figure 1).

### 2.2. Patient Positioning and Anesthetic Preparation

Following induction of general anesthesia, the patient is positioned for the robotic setup. A transesophageal echocardiography (TEE) probe is placed, and double-lumen endotracheal intubation is performed in all patients. Under TEE guidance, a venous cannula and a central venous catheter are placed in the right internal jugular vein, with the activated clotting time targeted at 200 s at this point. Robotic trocar docking sites and the thoracotomy site are marked on the skin according to the preoperative CT plan.

### 2.3. Robotic Docking

After surgical draping, the robotic trocars are positioned. In our current practice, the right robotic trocar is placed through the third; the camera port through the fifth; and the left robotic trocar through the seventh intercostal space. This configuration, instead of using the second, fourth and sixth intercostal spaces, was adopted in more recent cases to minimize collision with the left shoulder. When bilateral mammary artery harvest is planned, the ports may be positioned slightly more medially. The right working port later serves as the access site for the Chitwood clamp.

### 2.4. Robotic LIMA Harvest

Guided by the LIMA topography defined on preoperative CT, the LIMA is harvested with careful attention to any tortuosity. The endothoracic fascia is dissected anterior and posterior to the vessel down to the level of the bifurcation. Once the entire length of the LIMA has been mobilized, the patient is fully heparinized for CPB. The LIMA is clipped distal to the bifurcation using laparoscopic endoclips and divided. Hemostasis along the thoracic wall is checked under robotic visualization, after which robotic trocars are undocked.

### 2.5. Robotic RIMA Harvest

**Figure 2:**
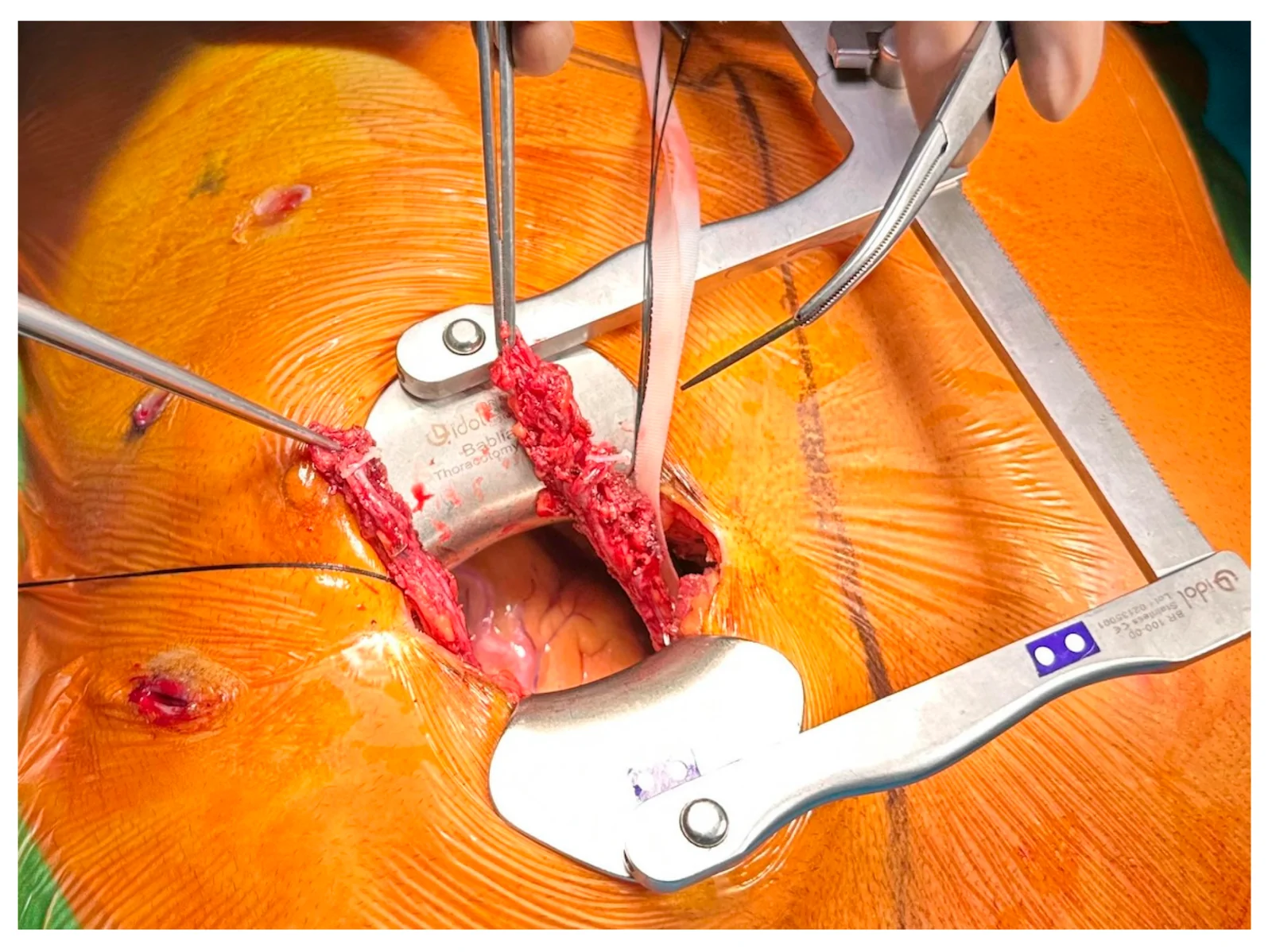
The full length of LIMA and RIMA can be harvested with robotic assistance.

When a bilateral internal mammary artery (BIMA) harvest is planned, the RIMA is harvested before LIMA from the right endothoracic fascia. The robotic arms are retracted to the left thorax for safe exchange during RIMA harvest. The harvest technique is similar to that of the LIMA. Safe dissection of the proximal RIMA can be challenging and may require gentle pressure on the heart for exposure. When adequate exposure cannot be achieved, the proximal segment may instead be completed on CPB after LIMA harvest (Figure 2).

### 2.6. Cannulation, Thoracotomy, and Conduit Harvest

Peripheral cannulation, either axillary or femoral, is then performed under TEE guidance. When any atherosclerotic plaque or thrombus is present at any level of the descending aorta or iliofemoral vessels, axillary cannulation is preferred to reduce the risk of retrograde thromboembolism and stroke. For direct axillary cannulation, an incision is made over the deltopectoral groove with ultrasound guidance. The axillary artery is identified and encircled with vascular tape, a 5-0 Prolene purse-string suture is placed, and the artery is cannulated directly by the Seldinger technique under TEE guidance. Femoral cannulation, when chosen, is performed analogously under TEE guidance through a 2 to 3 cm incision. A second surgeon performs the anterior minithoracotomy concurrently with peripheral cannulation. Additional conduits, saphenous veins or the radial artery, are prepared simultaneously at this stage using endoscopic harvesting.

### 2.7. Pericardiotomy and Aortic Cross-Clamping

**Figure 3:**
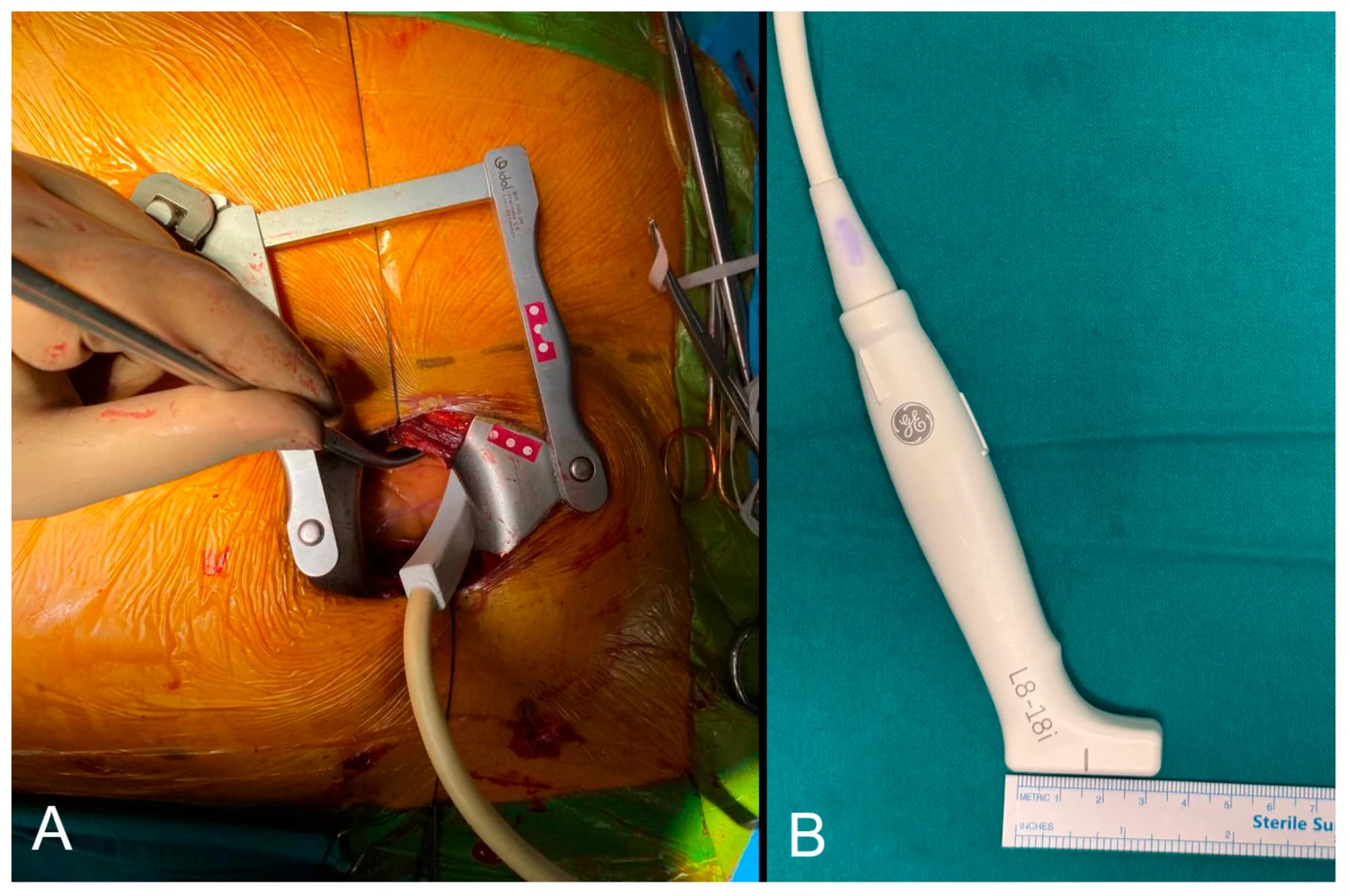
(**A**) The epiaortic ultrasound probe can be easily introduced through the minithoracotomy. (**B**) The “hockey-stick” probe (L8-18i-RS linear array transducer, GE HealthCare, Chicago, IL, USA) used for epiaortic ultrasonography.

The heart is decompressed by initiating CPB before the pericardium is opened. The pericardium is incised, the pulmonary artery and aorta are separated, and a Dacron tape is passed around the aorta. The cardioplegia root cannula is placed, and the Chitwood clamp is introduced through the right robotic trocar site. If there are suspected soft or calcific plaques in the ascending aorta, the L8-18i-RS linear array transducer (GE HealthCare, Chicago, IL, USA) is introduced through the minithoracotomy and an epiaortic ultrasound is performed to guide the placement of the Chitwood clamp and later the side-biting clamp for proximal anastomosis (Figure 3) (Supplemental Video S1). The heart is arrested with cardioplegia delivery. A posterior pericardial window is created to reduce postoperative effusion and the risk of atrial fibrillation.

### 2.8. Left Atrial Appendage Occlusion

When LAA occlusion is planned, the absence of thrombus within the left atrium and appendage is first confirmed by intraoperative TEE. Following cross-clamping and cardioplegia delivery, the AtriClip Flex Device (AtriCure, Inc., Mason, OH, USA) is introduced through the minithoracotomy and placed at the base of the appendage. Successful closure is confirmed with TEE (Supplemental Video S2). Patients received low-molecular-weight heparin and aspirin in the early postoperative period and a combination of direct oral anticoagulant and aspirin in the long term.

### 2.9. Exposure of the Target Vessels

The superior pulmonary veins and the inferior vena cava are encircled with tape to optimize distal exposure and facilitate the distal anastomoses technique described by Babliak et al. [17]. The coronary targets are evaluated, and the anastomotic sites are examined. Exposure of the distal coronary targets is obtained by traction on the encircling tapes.

### 2.10. Distal and Proximal Anastomoses

**Figure 4:**
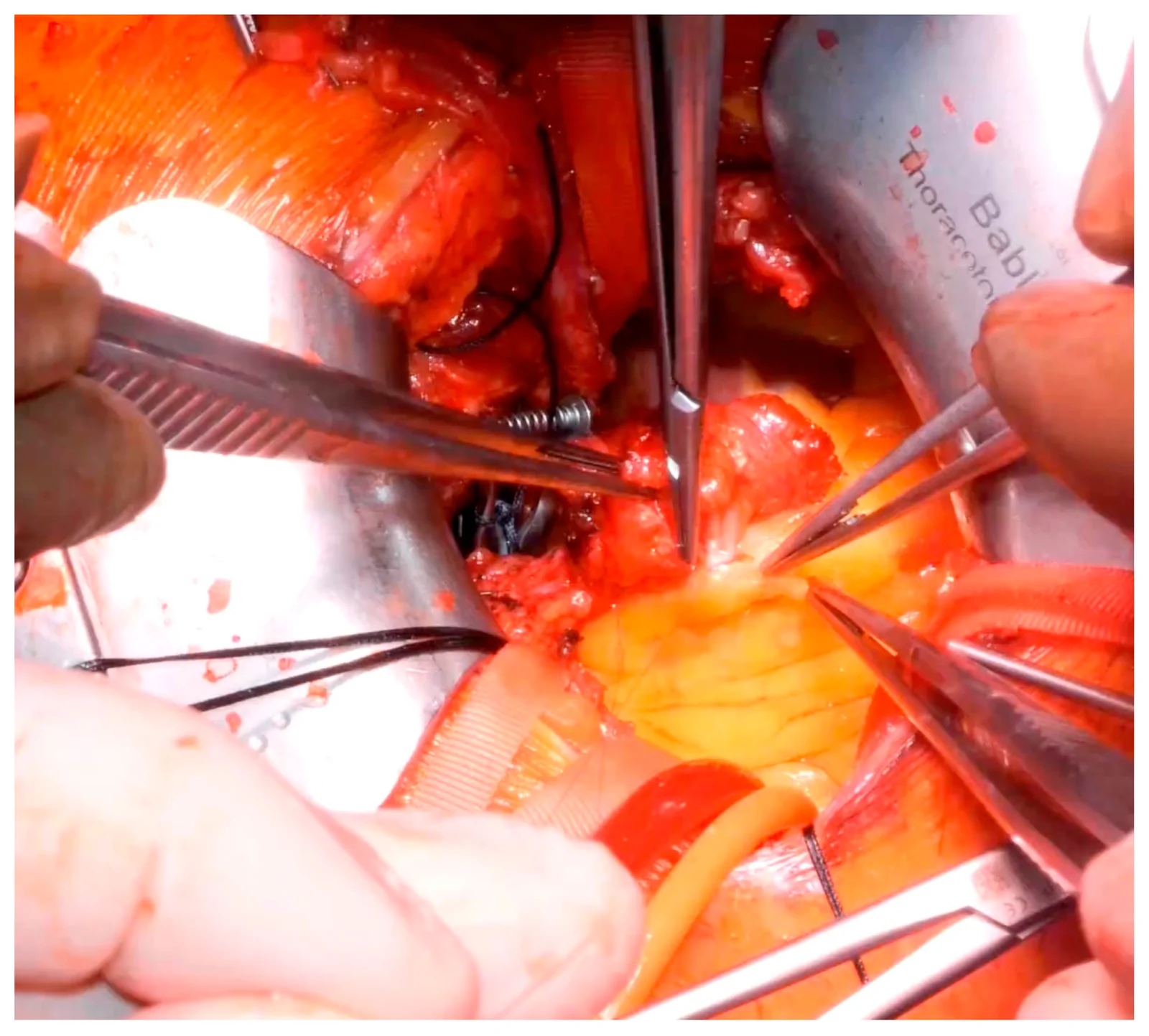
The RIMA is anastomosed to the RCA target through the minithoracotomy at the 3rd intercostal space on the arrested heart.

Distal anastomoses are performed under direct vision using the standard coronary anastomotic technique (Figure 4). This exposure is also sufficient to perform coronary endarterectomy and patch angioplasty when required. After completion of the distal anastomoses, the cross-clamp is removed, and a side-biting clamp is placed on the ascending aorta for the proximal anastomoses. The third or second intercostal access permits completion of the proximal anastomoses with minimal traction and aortic manipulation. Saphenous vein grafts are anastomosed proximally to the aorta; radial artery grafts are anastomosed to the aorta when suitable or otherwise to the LIMA (Supplementary Video S3). Patients were routinely maintained on dual antiplatelet therapy with clopidogrel 75 mg and aspirin 100 mg, including those who underwent endarterectomy.

### 2.11. Weaning and Closure

Transit-time flow measurement is performed after completion of all anastomoses to verify the quality of revascularization. Hemostasis is assessed at multiple stages during the operation: along the LIMA bed after harvest and before undocking the robot; at all distal and proximal anastomoses on CPB; and at the LIMA and RIMA, port sites, and chest wall under endoscopic visualization before weaning off CPB. The thoracic drain is placed before weaning from CPB through the left robotic trocar site. The thoracotomy is then closed. The patient is transferred to the intensive care unit, and autotransfusion is continued in the early postoperative period.

### 2.12. Statistical Analysis

Descriptive statistics were used to summarize patient demographics and surgical outcomes. Statistical analyses were performed using IBM SPSS Statistics version 25.0 (IBM Corp., Armonk, NY, USA) and R version 4.3.1 (R Foundation for Statistical Computing, Vienna, Austria) using the survival and ggsurvfit packages. The normality of the distribution of continuous variables was assessed using the Shapiro–Wilk test. Normally distributed continuous variables are presented as the mean ± standard deviation and variables without normal distribution are presented as the median and interquartile range. Normally distributed continuous variables were compared using Student’s *t*-test. Mid-term survival was estimated using the Kaplan–Meier analysis, measured from the date of operation to death from any cause. A *p*-value < 0.05 was considered statistically significant.

## 3. Results

Between January 2022 and June 2026, 242 patients underwent robotic-assisted MIDCAB at our institution. The mean age was 63.4 ± 9.7 years, and 31 patients (12.8%) were female. Baseline demographic characteristics are summarized in Table 1.

A single-vessel bypass was performed in 25 patients. Three single-vessel patients received a RIMA graft to the right coronary artery (RCA), one of which was a reoperation following prior LIMA-to-LAD and saphenous vein-to-RCA grafting, in which a RIMA graft was anastomosed to the RCA. Fifteen patients were operated off-pump. The rest of the on-pump patients had a mean CPB time of 155.7 ± 46.0 min. In total, 212 patients underwent an operation on the arrested heart and their mean CC time was 66.9 ± 21.7 min. Operative data are summarized in Table 2.

Full arterial revascularization was achieved in 42 (17.4%) patients. Of these patients, 22 (9.1%) underwent multivessel CABG. Among these multivessel full-arterial cases, nine (3.7%) incorporated bilateral mammary grafts. The radial artery was used in 43 (17.8%) patients, 32 (13.2%) of which were full-arterial cases. Patients who underwent full arterial revascularization were younger than the remainder of the cohort (59.9 ± 10.1 vs. 64.6 ± 9.3, *p* = 0.001). The conduits used and their target territories are summarized in Table 3. Conduits were used sequentially in more than one territory in a patient when anatomically suitable.

Calcific plaques of varying severity were identified in the ascending aorta in 25 (10.3%) patients, and a further two (0.8%) patients had soft, non-calcific plaques. In all of these patients, intraoperative epiaortic ultrasound was performed to determine the optimal sites for cross-clamp placement and proximal anastomosis. A total of 17 coronary endarterectomies were performed in 16 patients (6.6%), comprising 10 LAD and seven non-LAD endarterectomies. One patient required endarterectomy of both the LAD and right coronary arteries. Patch angioplasty of the non-LAD arteries was performed using a saphenous vein patch when necessary. The LIMA was preferred for patch angioplasty of the LAD whenever its caliber was suitable; otherwise, a saphenous vein patch was used, and the LIMA was anastomosed onto the patch. Concomitant LAA occlusion was performed in three (1.2%) patients.

Postoperative outcomes are presented in Table 4. In-hospital mortality occurred in two (0.8%) patients: one due to postoperative COVID-19-related complications and one due to multiorgan failure in the intensive care unit. One patient sustained a minor neurological deficit that subsequently resolved, and no patient experienced a major neurological deficit.

**Figure 5:**
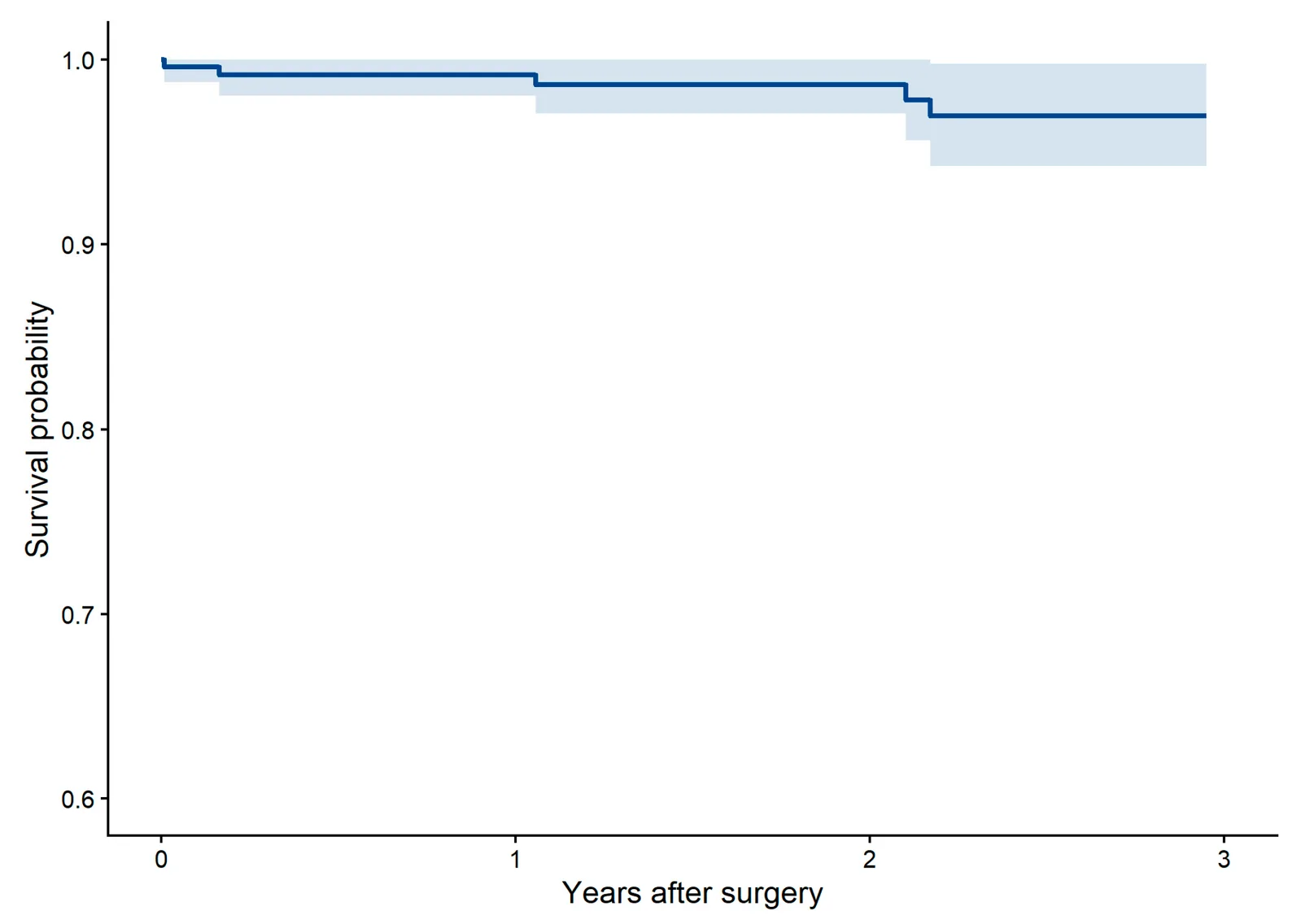
Kaplan–Meier plot of overall survival following robotic-assisted MIDCAB.

Median follow-up was 2.1 years. Two in-hospital deaths and three late deaths occurred among the study patients. Overall survival was 99.2% (95% CI 96.7–99.8) at 1 year and 97.0% (95% CI 92.5–98.8) at 3 years (Figure 5).

## 4. Discussion

Robotic-assisted minimally invasive direct coronary artery bypass offers advantages over conventional sternotomy CABG; however, it has been offered to a narrow patient population due to resource limitations, a steep learning curve, and patient selection that mostly includes single-vessel disease and patients with fewer comorbidities [19,20]. With accumulating institutional experience, these boundaries can be safely extended to encompass more patients with complex multivessel disease, including patients who require full-arterial revascularization, who have ascending aortic plaques, complex coronary disease requiring endarterectomy or atrial fibrillation where concomitant left atrial appendage occlusion is indicated, with low in-hospital mortality and morbidity.

Robotic-assisted MIDCAB renders complete and complex CABG feasible in patients who would otherwise require sternotomy, combining the conduit length afforded by robotic harvest with the proximal accessibility of a higher thoracotomy, while permitting distal anastomoses to all territories with the same ease as multivessel MIDCAB [21]. The robotic approach has clear advantages during mammary artery harvest. High-definition three-dimensional visualization and articulated instrumentation permit dissection of a longer LIMA segment with less chest wall trauma than direct-vision MIDCAB. The choice of thoracotomy site is a further point of distinction from conventional MIDCAB. Direct-vision MIDCAB favors the fourth or fifth intercostal space to obtain adequate LIMA length, occasionally requiring a second thoracotomy for proximal anastomosis, as described in some series [22]. The robotic harvest decouples conduit length from the thoracotomy level, permitting access through the third or second intercostal space [12,18]. This is advantageous for proximal anastomoses where traction on the aorta makes a lower incision hazardous. The coronary targets can be reached with gentle manipulation of the arrested, decompressed heart through a higher incision, and the third or second intercostal space approach minimizes aortic handling while facilitating side-biting clamp application. Anastomosis of a radial artery to LIMA, creating a T-graft, is likewise simplified at the upper intercostal spaces, making the use of the radial artery graft as a second arterial graft less technically demanding.

The robotic mammary harvest is particularly well suited to BIMA harvest, allowing the RIMA to be taken down to reach diverse coronary targets in a safe and reproducible fashion, with the BIMA configuration tailored to individual anatomy [23]. While there is a learning curve to robotic RIMA harvest, its application can allow RA-MIDCAB in patients who are planned to undergo full arterial revascularization with bilateral mammary arteries. The exposure achieved with the described technique is sufficient for complete revascularization with any number of distal anastomoses. In our experience, the arrested-heart field obtained through the second or third intercostal spaces permits multivessel grafting with up to six distal anastomoses, as well as additional procedures such as coronary endarterectomy with patch angioplasty. In patients with a concomitant indication, left atrial appendage occlusion can be performed under transesophageal echocardiographic guidance during the same procedure, broadening the operation’s applicability in those with atrial fibrillation [24].

Management of the atherosclerotic ascending aorta is integral to extending this approach to patients with more complex atherosclerotic disease. Epiaortic ultrasonography through the minithoracotomy guides the clamp placement and proximal strategy, consistent with guideline recommendations for intraoperative aortic assessment to mitigate embolic stroke [2,25]. In the presence of a heavily calcified aorta, beating heart or total arterial revascularization asanaortic strategies may also be considered to avoid aortic manipulation altogether.

The 25-year review by Bonatti et al. encompasses minimally invasive and robotic CABG performed with several techniques [4]. RA-MIDCAB represented 15.8% of the reviewed cases with 1762 patients. In this group, 85.5% of procedures were single-vessel LIMA-to-LAD grafting, with a mean of 1.3 grafts per patient, and mortality and stroke rates were 0.4%. Our technique combines the advantages of robotic mammary harvest with full multivessel revascularization, achieving a mean of 2.8 distal anastomoses with comparably low mortality (0.8%) and no major stroke. Survival in our cohort compares favorably with the long-term data available for robotic-assisted MIDCAB [10,26].

The main strength of this study is that it represents a consecutive series of full revascularization with robotic-assisted MIDCAB, including patients undergoing total arterial revascularization, bilateral internal mammary artery grafting, coronary endarterectomy, aortic plaque management guided by epiaortic ultrasonography, and concomitant left atrial appendage occlusion. The procedures were performed with a standardized technique by a single surgical team, which eliminated inter-surgeon variability. Certain limitations of this study exist. It is a single-center observational study without a comparison group and conclusions can be drawn only on feasibility and safety, not on superiority. While mid-term survival data were reported, no routine postoperative angiographic or computed tomographic assessment was performed, and no conclusions can be made regarding graft patency.

Robotic-assisted coronary surgery is likely to play an increasingly important role in surgical revascularization with structured training and careful program development [27]. Although the well-documented learning curve remains a principal constraint, adoption is expanding beyond pioneering centers [28,29]. The evidence base for non-sternotomy multivessel CABG is growing, as demonstrated by the recent MIST trial, and future trials incorporating robotic assistance, routine graft surveillance, and patient-reported outcomes will help define its efficacy relative to sternotomy CABG and hybrid revascularization strategies [5]. Within this evolving landscape, the approach described here may extend the reach of RA-MIDCAB to patients who would otherwise be denied a sternal-sparing operation. Nevertheless, there are certain groups of patients for whom this approach is still not routinely suitable, including reoperative cases and urgent revascularization. One patient in our cohort underwent a redo operation for RCA bypass using the RIMA through a right minithoracotomy. A small number of redo robotic-assisted CABG cases have been reported with satisfactory outcomes [30]. With more experience, RA-MIDCAB can be applied to select redo cases to benefit more patients.

## 5. Conclusions

With careful planning and accumulated experience, the indications for robotic-assisted minimally invasive revascularization can be extended to complete multivessel CABG in a broader patient population. Patients who require full-arterial revascularization, have ascending aortic plaques, complex coronary disease requiring endarterectomy or atrial fibrillation where concomitant left atrial appendage occlusion is indicated can benefit from this approach, with low in-hospital mortality and no major stroke.

## Figures and Tables

**Table 1:** Patient demographics.

	*n* = 242
Age	63.4 ± 9.7
Female Gender	31 (12.8%)
Body Mass Index	28.7 ± 4.3
Smoker	168 (69.4%)
Diabetes Mellitus	112 (46.3%)
Hypertension	153 (63.2%)
Hyperlipidemia	172 (71.1%)
Previous Myocardial Infarction	32 (13.2%)
Previous Percutaneous Coronary Intervention	59 (24.4%)
Chronic Obstructive Pulmonary Disease	6 (2.5%)
Left Ventricular Ejection Fraction	58.3 ± 6.9
EuroSCORE II	2.12 ± 1.89

**Table 2:** Operative data.

	*n* = 242
Procedure	
Off-Pump	15 (6.2%)
On-Pump Beating	15 (6.2%)
Cross Clamp	212 (87.6%)
Distal Anastomosis	2.8 ± 1.1
Proximal Anastomosis	1.1 ± 0.7
CPB ^1^ Time (min)	155.7 ± 46.0
Cross-Clamp time (min)	66.9 ± 21.7

^1^ CPB: Cardiopulmonary bypass.

**Table 3:** Conduits and their anastomosis counts to target territories.

Target Artery	LIMA (*n* = 263)	RIMA (*n* = 12)	Radial Artery (*n* = 57)	Saphenous Vein (*n* = 304)
LAD	236 (89.7%)	3 (25.0%)	0 (0%)	3 (1.0%)
Diagonal	22 (8.4%)	0 (0%)	6 (10.5%)	63 (20.7%)
Intermediate	1 (0.4%)	0 (0%)	3 (5.3%)	14 (4.6%)
Circumflex	2 (0.8%)	1 (8.3%)	32 (56.1%)	125 (41.1%)
RCA	1 (0.4%)	8 (66.7%)	16 (28.1%)	99 (32.6%)

LAD: left anterior descending artery; LIMA: left internal mammary artery; RCA: right coronary artery; RIMA: right internal mammary artery.

**Table 4:** Operative outcomes.

	*n* = 242
Mechanical ventilation time (hours)	4.0 (3.0–6.0)
Intensive care unit stay (hours)	22.6 ± 11.2
Transfusion requirement	24 (9.9%)
Reoperation for bleeding	2 (0.8%)
Conversion to sternotomy	0 (0%)
Minor stroke	1 (0.4%)
Major stroke	0 (%)
Mortality	2 (0.8%)

## Data Availability

The data supporting the findings of this study are available from the corresponding author upon request.

## Supplementary Materials

The following supporting information can be downloaded at https://www.mdpi.com/article/10.3390/jcdd13090411/s1. Video S1: Epiaortic ultrasound on the ascending aorta with calcific plaques. Video S2: Concomitant left atrial appendage closure. Video S3: Proximal anastomosis of saphenous graft to the aorta.

## Abbreviations

The following abbreviations are used in this manuscript:

BIMA	Bilateral internal mammary artery
CABG	Coronary artery bypass grafting
CPB	Cardiopulmonary bypass
CT	Computed tomography
LAA	Left atrial appendage
LAD	Left anterior descending
LIMA	Left internal mammary artery
MIDCAB	Minimally invasive direct coronary artery bypass
RA-MIDCAB	Robotic-assisted minimally invasive direct coronary artery bypass
RCA	Right coronary artery
RIMA	Right internal mammary artery
TEE	Transesophageal echocardiography
